# Successful conversion surgery for locally advanced gallbladder cancer after gemcitabine and nab-paclitaxel chemotherapy

**DOI:** 10.3389/fonc.2022.977963

**Published:** 2022-08-16

**Authors:** Ziyi Yang, Ziyou Wu, Yichen Xiong, Shilei Liu, Chen Cai, Ziyu Shao, Yidi Zhu, Xiaoling Song, Wei Shen, Xuefeng Wang, Xiangsong Wu, Wei Gong

**Affiliations:** ^1^ Department of General Surgery, Xinhua Hospital Affiliated to Shanghai Jiao Tong University School of Medicine, Shanghai, China; ^2^ Shanghai Key Laboratory of Biliary Tract Disease Research, Shanghai, China; ^3^ Good Clinical Practice (GCP) Office, Xinhua Hospital Affiliated to Shanghai Jiao Tong University School of Medicine, Shanghai, China

**Keywords:** gallbladder cancer, resectability, gemcitabine, nab-paclitaxel, conversion therapy

## Abstract

**Objective:**

Gallbladder cancer (GBC) is highly malignant and is often diagnosed at the advanced stage. Lack of opportunity to surgery results in an unsatisfactory outcome. This pilot study employed gemcitabine combined with nab-paclitaxel (AG) as a conversion therapeutic measure for locally advanced GBC and successfully achieved conversion surgery in three initially unresectable GBC patients. We will introduce our experience on improving the outcome of this dismal disease.

**Methods:**

Radiology and nuclear medicine imaging were performed in each patient, and resectability was evaluated by joint consultation of our multi-disciplinary team (MDT). Patients evaluated as unresectable were treated with the AG regimen and re-evaluated for treatment response. When complete or partial response is achieved, MDT opinion would be required to assess the possibility of performing conversion surgery with R0 resection.

**Results:**

Three GBC patients who were initially evaluated as unresectable successfully underwent R0 resection after conversion therapy with the AG regimen. The first case was a recurrent GBC patient evaluated as locally advanced and eventually achieved pathological complete response. The second case was a GBC patient who underwent R1 resection with residual lesions in the gallbladder bed and isolated No. 16 lymph node metastasis and who had a pathologically complete response after treatment. The third case had multiple but resectable liver metastases; both objective response and partial pathologic response were achieved. None of the patients experienced serious treatment-related adverse events. All cases revealed no evidence of recurrence or metastasis after a median follow-up of 12 months.

**Conclusions:**

Conversion therapy shows a favorable efficacy in those unresectable GBC patients. Gemcitabine plus nab-paclitaxel has the potential to be used as a preoperative treatment option for GBC patients at the advanced stage. To further explore the efficacy of AG on conversion therapy for GBC patients, a prospective clinical trial has been registered (ChiCTR2200055698).

## Introduction

Gallbladder cancer (GBC) is one of the most lethal cancers, with reports of rising incidence and mortality rates (1, 2). Due to the lack of effective systemic treatments, surgery offers the only chance for cure. However, GBC usually develops with insidious onset. More than 50% of patients are already at an advanced stage when the tumor is detected, making radical surgical resection difficult or even impossible (3), while the recurrence rate after surgery is not low (4). At present, palliative chemotherapy is usually considered to be the treatment option for unresectable GBC, but shows a significantly worse prognosis compared to resected GBC (5). Therefore, it is very important to create surgical opportunities for GBC patients and to raise the proportion of radical surgery to improve the outcome.

Conversion therapy aims to achieve a downstaging effect through systemic therapy, with the expectation of radical surgical resection for previously unresectable tumors. This treatment pattern offers a new paradigm for advanced solid tumors and has made progress in liver, gastric, and colorectal cancers (6–8). Thus, it is necessary to establish a conversion therapy framework for GBC (9). Specifically, the following three issues need to be addressed: (1) the resectability assessment of GBC; (2) the modalities of systemic therapy; and (3) the timing of conversion surgery.

**Figure 1:**
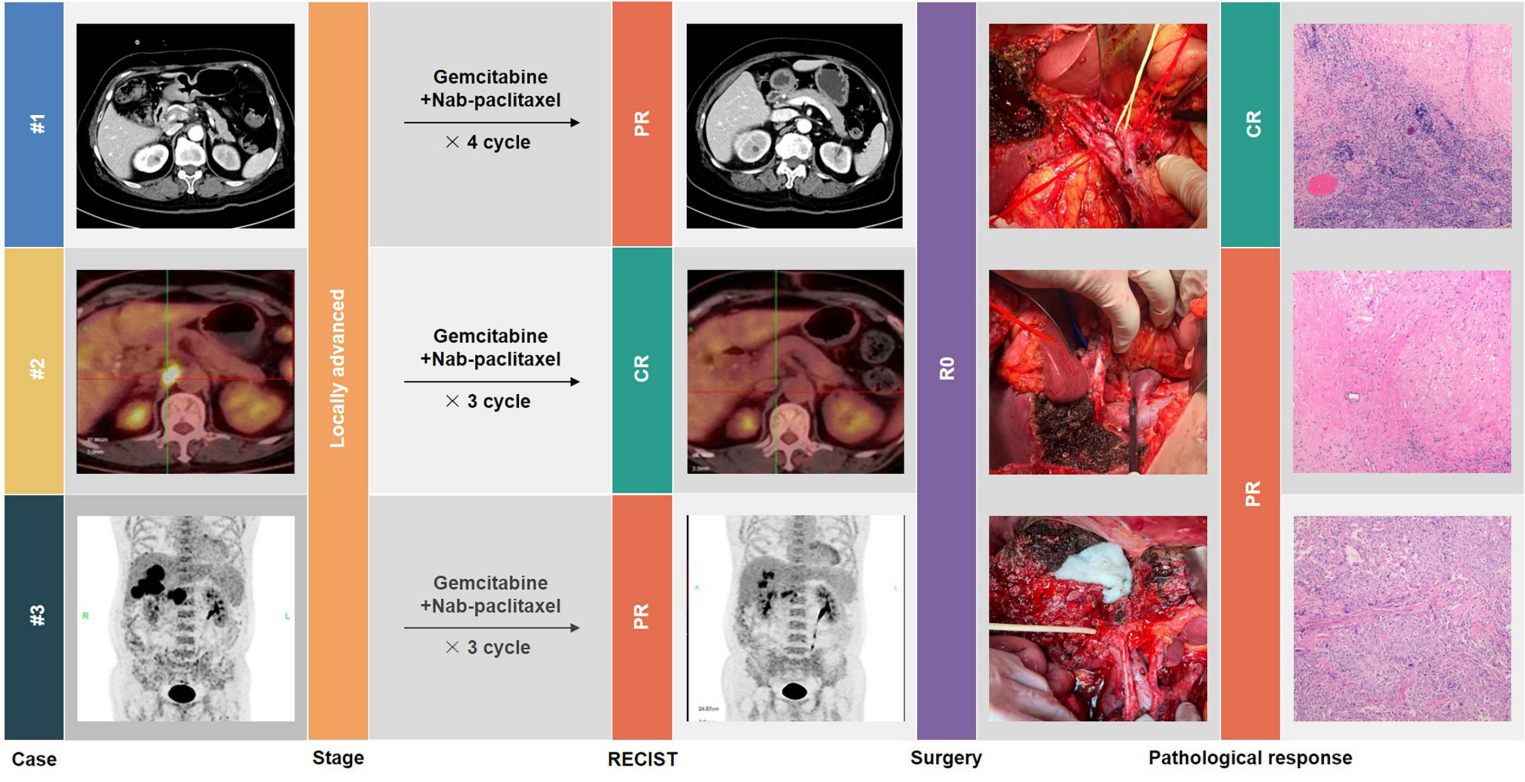
Graphical abstract.

This article describes our experience in the conversion therapy of GBC. Through three typical cases with successful conversion surgery (**Figure 1**), the Xinhua criteria for assessing resectability of GBC, and the effectiveness of gemcitabine plus nab-paclitaxel (AG) as a chemotherapy regimen are discussed in detail. We believe that this could provide a new possibility for conversion therapy of GBC.

## Methods

### Patients and data collection

We reviewed patients diagnosed with pathologically confirmed GBC in our center from January 2020 to December 2021; three GBC patients with initial unresectable status who successfully underwent R0 resection after AG chemotherapy were included.

Patient information was completely collected, including baseline demographics and clinical characteristics, tumor characteristics, systemic therapy records, biological examinations, changes in imaging examinations, and surgery records. Clinical outcomes, such as postoperative complications, recurrences, and survival data, were also collected.

### Resectability assessment

**Figure 2:**
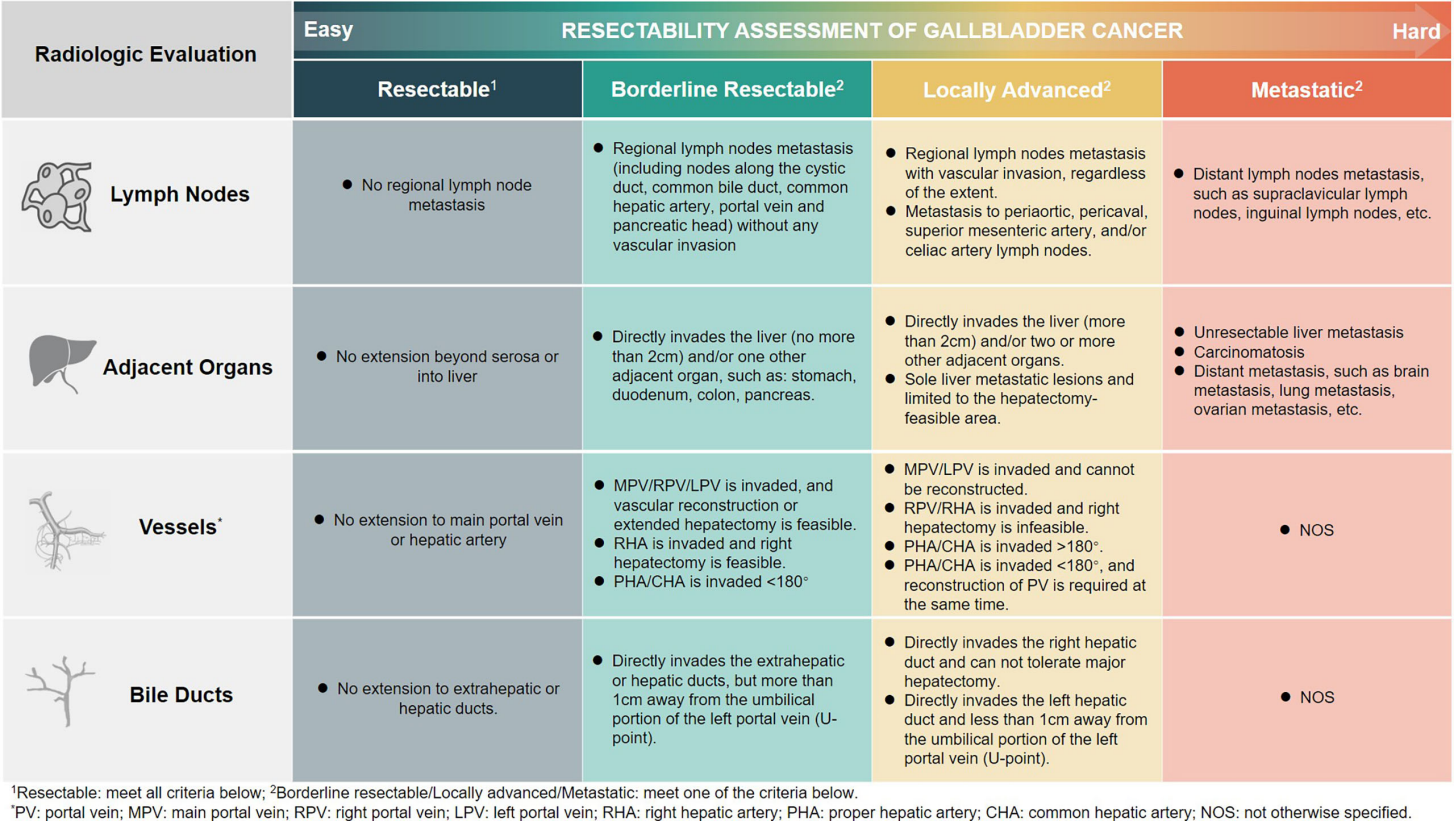
The resectability assessment of gallbladder cancer (Xinhua criteria).

The resectability assessment was performed using radiology and nuclear medicine imaging methods. We classified GBC into four subgroups (Xinhua criteria), namely, resectable, borderline resectable, locally advanced, and metastatic, with decreasing resectability and progressively worse prognosis. Lymph nodes, adjacent organs, blood vessels, and bile ducts are the main aspects of our evaluation. The detailed information is shown in **Figure 2**.

### Chemotherapy and toxicity assessment

Unresectable locally advanced and metastatic GBC patients were treated with AG as conversion therapy. The treatment decision was made by the multi-disciplinary team (MDT) following the principle of evidence-based medicine and was approved by the Ethics Committee of Xinhua Hospital affiliated to Shanghai Jiao Tong University School of Medicine. In addition, the treatment was conducted in accordance with the Declaration of Helsinki and principles of Good Clinical Practice. Written informed consent of all patients was obtained.

All patients were infused on the first and eighth day of a 21-day cycle and received an initial dosage of nab-paclitaxel, 125 mg/m^2^, followed by gemcitabine, 1,000 mg/m^2^, each for 30 min of intravenous injection, which was personalized according to the patient’s condition (10).

All toxicity adverse reactions were classified according to NCI-CTC 2.0. Adverse events of patients were closely observed during medication. If a toxicity of grade 3 or above was observed, the medication should be stopped immediately, and the dose should be reduced according to the specific situation after returning to the normal value.

### Efficacy evaluation

The hematological examination was performed before each medication, and an imaging examination was performed every 4 weeks. After three to four cycles, according to Response Evaluation Criteria in Solid Tumors 1.1 (RECIST 1.1) criteria, the multidisciplinary team evaluated the response to the treatment and discussed the possibility of potential R0 resection.

### Conversion surgery

When complete or partial response is achieved, MDT opinion would be required to assess the possibility of performing conversion surgery with R0 resection. The extent of resection was determined on a case-by-case basis. For GBC that still presented with primary lesions, radical cholecystectomy and regional lymph node dissection must be completed. As to the extent of liver resection, at least anatomical segment IVb/V resection would be performed if there was significant invasion of the liver. For GBC that needs re-resection, wedge resection of the gallbladder bed and regional lymph node dissection would be performed at least.

### Postoperative adjuvant therapy and follow-up

All patients who underwent conversion surgery received a four-cycle adjuvant chemotherapy with gemcitabine and nab-paclitaxel, using the same dosage as preoperative chemotherapy. The adjuvant treatment also obtained the approval from the Ethics Committee of Xinhua Hospital affiliated to Shanghai Jiao Tong University School of Medicine and the written informed consent from all patients.

Patients were followed up by laboratory tests including tumor markers once a month and imaging examinations once every 3 months to monitor potential recurrence or metastasis.

## Results

### Conversion therapy of recurrent GBC

The first patient, a 66-year-old woman, was admitted to another hospital in August 2018 for “gallbladder stones” and underwent laparoscopic cholecystectomy, with a postoperative pathological diagnosis of gallbladder adenocarcinoma (grade II–III), infiltrating into the deep muscular layer (T1b). However, she did not receive further radical surgery or any adjuvant treatment. When she came to our outpatient clinic in March 2020, CT enhancement of the abdomen suggested enlarged lymph nodes in the hepatoduodenal ligament, invading the main trunk of the portal vein (PV) and the posterior wall of the common hepatic artery (CHA). PET/CT showed FDG-avid lymph nodes adjacent to the portal vein, and metastasis was first considered, with no obvious distant metastasis. There were no significant abnormalities in blood routine, liver function, and coagulation function; carcinoembryonic antigen (CEA) was 16.54 ng/ml, CA72-4 was 17.80 U/ml, and the other tumor marker levels were in the normal range. The diagnosis of regional lymph node recurrence of incidental gallbladder cancer was established.

According to our criteria of resectability assessment, when the main PV and CHA are invaded and cannot be reconstructed, the cancer should be classified to unresectable locally advanced stage. AG chemotherapy was started following MDT discussion. After four cycles, enhanced CT of the abdomen showed significant shrinkage of the target lymph nodes, which approximately disappeared, the periportal space of the portal vein and common hepatic artery became clear, and the tumor markers decreased to the normal range, achieving clinical partial response (PR) according to RECIST 1.1 criteria, with the opportunity for radical surgery. Radical regional lymphadenectomy with No. 16 lymph node sampling, wedge resection of the gallbladder bed, and resection of the extrahepatic bile duct were performed. Postoperative pathology showed negative bile duct margins and no tumor residue in the gallbladder bed, and a total of 13 lymph nodes were harvested, including the No. 7, No. 8, No. 12, No. 13, and No. 16 lymph nodes, all of which were reactive hyperplasia. One of the lymph nodes had fibrous tissue hyperplasia with histiocytic reaction, suggesting post-treatment changes of the tumor. Pathological complete response was achieved.

**Figure 3:**
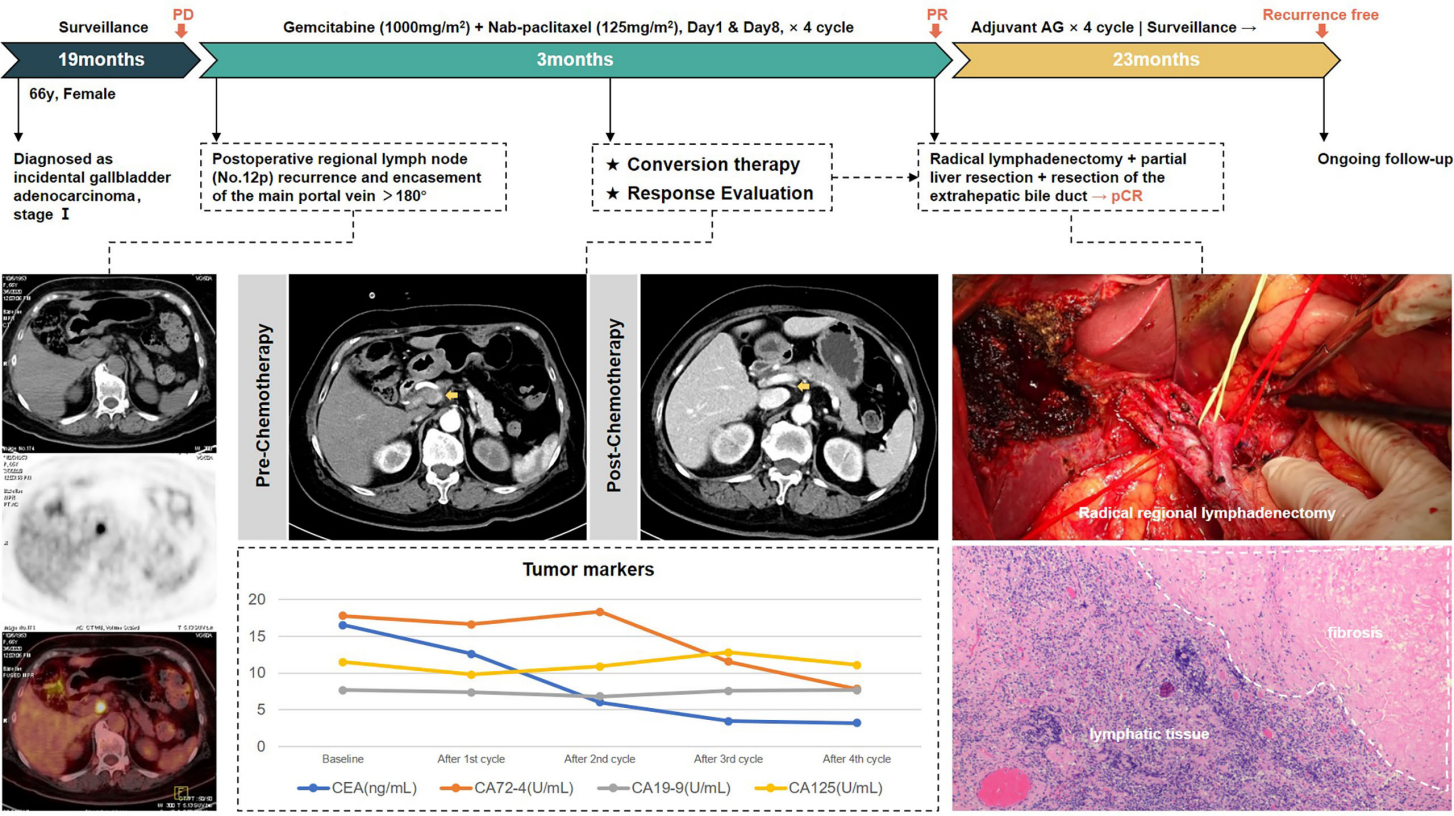
The treatment process of patient #1.

The patient received four cycles of adjuvant AG regimen. There was no sign of tumor recurrence at 23 months of follow-up so far. Treatment-related adverse events during the entire process included grade 2 leukopenia and nausea. The whole process of treatment is presented in **Figure 3**.

### Conversion therapy of GBC with distant lymph node metastasis

The second case was a 67-year-old woman with hypertension and diabetes. She presented to another hospital in March 2021 with abdominal pain. CT scan showed gallbladder stones with chronic cholecystitis. After undergoing laparoscopic cholecystectomy, pathology examination showed gallbladder adenosquamous carcinoma invading to the seroma, with vascular and nerve infiltration. Moreover, the margins of the specimen were positive. After admission to our hospital, imaging evaluation using PET/CT revealed residual lesions in the gallbladder bed with metastasis in the periaortic lymph nodes (No.16a_2_). CEA and CA-125 were above normal range.

In accordance with previous experience, periaortic lymph nodes are classified as distant lymph nodes. However, based on MDT discussion, we considered that for gallbladder cancer, isolated No. 16a2 lymph node metastasis still had the possibility of R0 resection and was therefore classified as locally advanced. After three-cycle AG chemotherapy, the metastatic lymph nodes exhibited a major response, not only in the regressed size, but also in the disappearance of FDG uptake. Then, she received anatomical live resection of segment IVb/V and extended lymphadenectomy including periaortic lymph node dissection. R0 resection was successfully achieved. Postoperative pathology showed adenocarcinoma involvement in the fibrous tissue of the gallbladder fossa and no tumor involvement in the liver parenchyma. A total of 16 lymph nodes were harvested; only one lymph node in the No. 13 group was positive and five lymph nodes in the No. 16 group were negative, implying a complete pathological response of the periaortic metastases.

**Figure 4:**
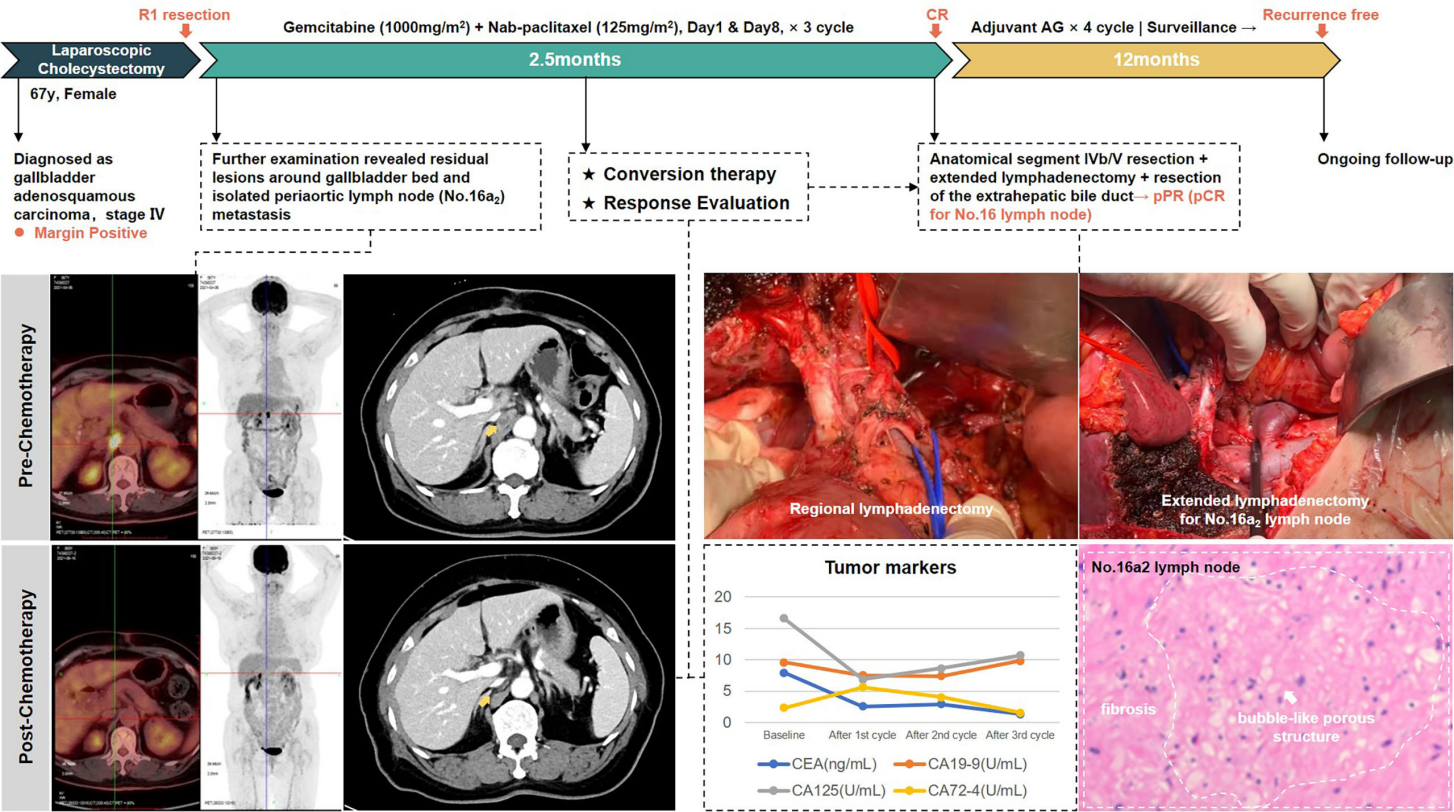
The treatment process of patient #2.

This patient received four cycles of gemcitabine plus nab-paclitaxel as postoperative adjuvant chemotherapy. After 12 months of follow-up to date, she maintains a relapse-free status. No ≥grade 3 treatment-related adverse events occurred during the entire process. The whole process of treatment is presented in **Figure 4**.

### Conversion therapy of GBC with liver metastasis

The third patient was a 70-year-old man. He presented to our department in April 2021 with poor appetite and bilateral back radiating pain over the last 2 months. The abdominal enhancement CT scan and PET/CT scan indicated gallbladder malignancy, with the adjacent liver parenchyma invaded and multiple intrahepatic metastases, the larger one of which was 3.9 × 3.4 cm in size located in the anterior upper segment of right hepatic lobe. In addition, multiple enlarged lymph nodes in the hilar region and retroperitoneum were considered metastasis. Liver biopsy then confirmed gallbladder adenocarcinoma. The diagnosis of metastatic GBC at stage IV was made.

This is an obviously unresectable case with poor prognosis, but surprisingly, the patient achieved partial response after three cycles of AG chemotherapy. Radiology examinations revealed significant remission of the primary lesion as well as liver and lymph node metastases. The levels of CA19-9 dropped from 1,329 U/ml to 68.3 U/ml. Therefore, he underwent extended radical surgery for gallbladder cancer, including cholecystectomy, meso-hepatectomy (segment IV/V/VIII), and both regional and extended lymphadenectomy. Postoperative pathology showed moderate to low differentiated adenocarcinoma of gallbladder, invading to adjacent liver parenchyma. Of 17 lymph nodes retrieved, 3 were positive for metastasis. Notably, the surgical margins were all negative and the patient recovered rapidly without postoperative complications.

**Figure 5:**
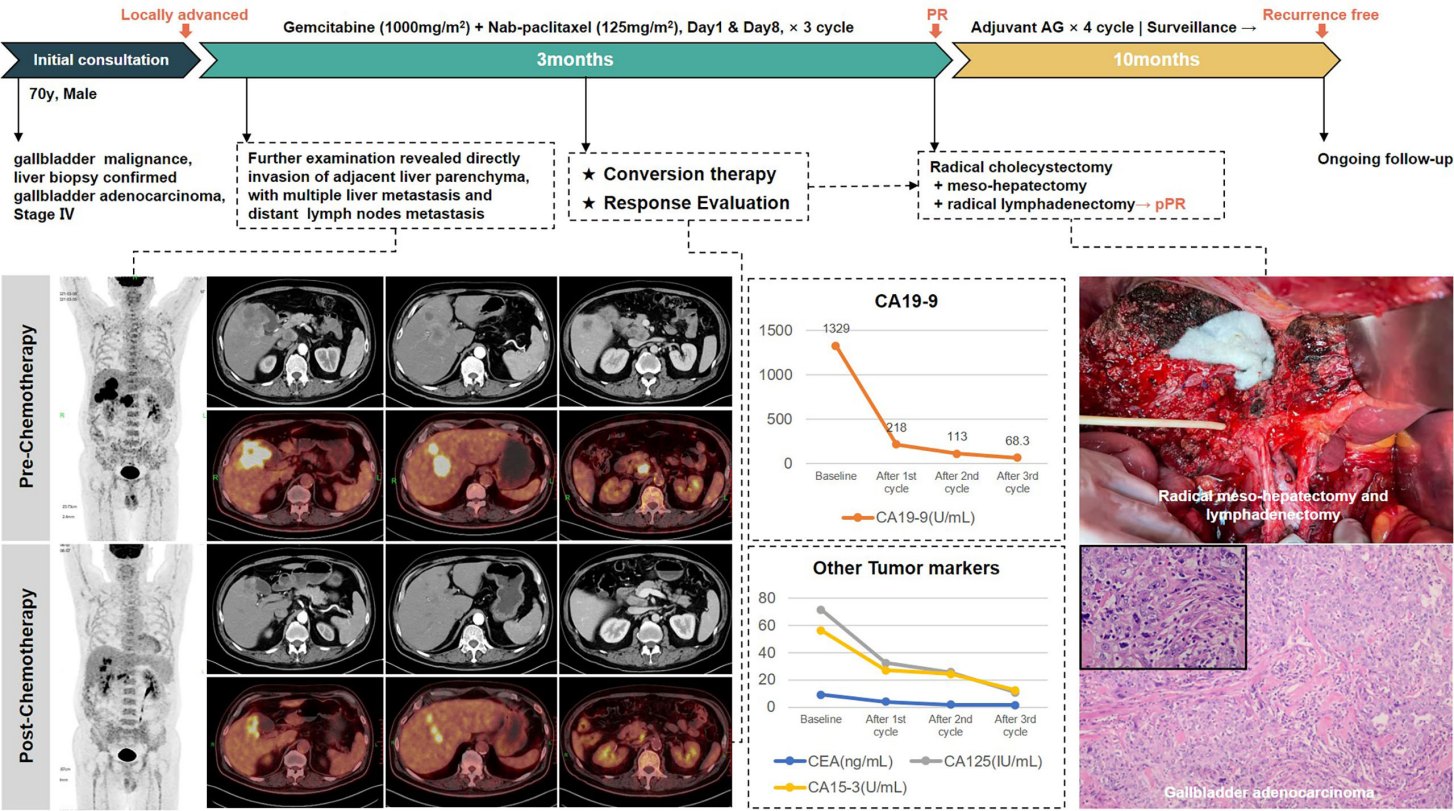
The treatment process of patient #3.

Four-cycle AG chemotherapy was added as postoperative adjuvant therapy. He had not shown signs of recurrence after 10 months of follow-up. Treatment-related adverse events during the entire process included grade 2 leukopenia and fatigue. The whole treatment process is presented in **Figure 5**.

## Discussion

Surgery is currently the only possible cure for GBC. Our previous study enrolled the largest multicenter cohort of GBC in China, with a total number of 6,159 patients in the registry (11, 12). We found that 34.26% of patients could not undergo surgery, and of those who received surgery, only 58.89% achieved radical resection. A significant difference could be found in the overall survival between non-radical resection and radical resection. The same conclusion that radical resection for GBC is associated with improved survival can also be found from the National Cancer Database in America (13). It indicates that radical surgery with the goal of R0 resection is particularly important to improve the prognosis of GBC, especially for GBC diagnosed at the advanced stage, which requires more accurate preoperative assessment of resectability.

GBC at the advanced stage is a challenge for surgeons because it is very unlikely to achieve R0 resection if upfront surgery is performed without a thorough and accurate preoperative evaluation by experienced MDT consultation. Furthermore, in some of the cases, even if extended surgery is performed, which simultaneously resected involved vessels and invaded organs to achieve clean margins, the long-term survival is still unsatisfactory. This is exemplified by the recent progress in the treatment of pancreatic cancer that defines pancreatic cancer into borderline resectable, locally advanced, and metastatic (14, 15). When evaluated as unresectable, whether technically unresectable or oncologically unresectable, the decision to upfront surgery should be avoided.

Current guidelines provide some principles on the indications for surgery, but offer limited help in clinical judgment of resectability (16, 17). In order to render a more practical and effective approach to assess the resectability of the GBC patient in clinical settings, we proposed the Xinhua criteria for the resectability of GBC at the advanced stage. The involvement of vital blood vessels, the invasion of neighboring organs, and the status of distant metastasis, mainly based on the anatomical features of both primary tumor and metastatic lesions, were the focus of attention. Medical imaging results are the key to our assessment (18, 19). Abdominal enhanced CT is most frequently used to determine the invasion of the tumor into surrounding blood vessels and adjacent organs. PET/CT is also important for the evaluation of tumor stage and to detect the presence of distant metastases. We further classified advanced GBC into four subtypes, which combined the information derived from radiological imaging findings and the feasibility of achieving en bloc resection. Therefore, limited resectable liver metastasis, which is traditionally classified as M1 in the TNM staging system but has benefited from surgery (20), is classified at the locally advanced stage.

Conversion therapy is suitable for unresectable GBC. Several reported cases have shown that unresectable GBC can be converted successfully to resectable GBC (21–24), which can greatly improve survival. However, the dosing regimens for conversion therapy are inconclusive. The first-line chemotherapy drugs for advanced GBC are gemcitabine plus platinum-based regimens, resulting from the ABC-02 trial in 2010 (25), with a median overall survival (OS) of 11.7 months and a median progression-free survival (PFS) of 8 months. This is not a satisfactory result. More importantly, the purpose of conversion therapy is to create an opportunity for surgery, requiring a high objective remission rate (ORR). Previous studies in unresectable biliary tract cancers reported that GC (gemcitabine and cisplatin) had an ORR of 16.2% (26), GEMOX (gemcitabine and oxaliplatin) had an ORR of 18.9% (27), and FOLFIRINOX (oxaliplatin, irinotecan, and 5-fluorouracil) had an ORR of 25% (28). Encouragingly, a recent phase II trial showed that the best ORR of AG in advanced cholangiocarcinoma was 30% (10), which may facilitate the implementation of radical surgery. Another clinical trial proved that nab-paclitaxel combined with gemcitabine and cisplatin prolonged PFS (11.8 months) and OS (19.2 months) in advanced biliary tract cancers compared to gemcitabine combined with cisplatin, while 16% patients withdrew owing to adverse events (29). Considering the treatment-related adverse events, we finally adopted a diphasic regimen of gemcitabine plus nab-paclitaxel.

The timing and approach to conversion surgery are individualized. In principle, the optimal surgical indication is to achieve oncological resectability. The extent of surgery included radical resection of the primary/secondary lesions and complete regional lymph node dissection. The extended lymph node dissection seems to provide a survival benefit (30, 31). Whether extrahepatic bile ducts should be routinely removed needs further studies (32). Long-term conversion therapy may affect organ function, increase the risk of surgical intervention, and cause tumor escape. Surgeons need to grasp the opportunity of conversion surgery. Tumor markers such as CEA and CA19-9 may provide some guidance, in addition to imaging examinations.

More efforts are still needed to overcome the limitations of the current conversion therapy paradigm. Firstly, this paper is designed to summarize the experience of conversion therapy for GBC by reviewing successful cases. In order to generate high-quality evidence, our center has launched a prospective trial (ChiCTR2200055698) to study the efficacy and safety of gemcitabine combined with nab-paclitaxel in the treatment of unresectable GBC. Secondly, we only use chemotherapy in preoperative treatment while a wider range of precise treatment modalities are available in the era of molecular targeted therapy and immune checkpoint inhibitors (33). Combination therapy needs to be studied in the future. Furthermore, we need to discover more reliable biomarkers to build efficacy prediction models that can better guide the selection of conversion treatment options.

In conclusion, this is the first reported case series of AG regimen on conversion therapy for locally advanced GBC. Our findings highlight that resectability assessment is essential and gemcitabine plus nab-paclitaxel is an effective preoperative treatment option for GBC patients at the advanced stage. Ongoing studies will accumulate more evidence to support our notion.

## Data availability statement

The original contributions presented in the study are included in the article/supplementary material. Further inquiries can be directed to the corresponding authors.

## Ethics statement

This study was reviewed and approved by The ethics Committee of Xinhua Hospital affiliated with Shanghai Jiao Tong University School of Medicine. The patients/participants provided their written informed consent to participate in this study. Written informed consent was obtained from the individual(s) for the publication of any potentially identifiable images or data included in this article.

## Author contributions

ZY, ZW and YX wrote the manuscript. SL, CC, ZS, YZ, XS, WS and XWang collected the clinical data of patients. ZY designed the figures. XWu and WG performed the critical review. All authors participated in the care of patients, contributed to the article and approved the submitted version.

## Funding

This work was funded by the National Natural Science Foundation of China (No. 82172628, 81974371, 82173048 and 81902864); Xinhua Hospital Funded Clinical Research (21XHDB10).

## Acknowledgments

We thank the patients and the staff in the Department of General Surgery of Xinhua Hospital.

## Conflict of interest

The authors declare that the research was conducted in the absence of any commercial or financial relationships that could be construed as a potential conflict of interest.

## Publisher’s note

All claims expressed in this article are solely those of the authors and do not necessarily represent those of their affiliated organizations, or those of the publisher, the editors and the reviewers. Any product that may be evaluated in this article, or claim that may be made by its manufacturer, is not guaranteed or endorsed by the publisher.
